# Supplementary material for: The Inhibitory Role of miR-486-5p on CSC Phenotype Has Diagnostic and Prognostic Potential in Colorectal Cancer
Source: Cancers (Basel). 2020 Nov 19;12(11):3432. doi: 10.3390/cancers12113432 (PMC7699298; doi:10.3390/cancers12113432)
Supplement: Supplementary file 1 [file cancers-12-03432-s001.pdf]

cancers

Article

The Inhibitory Role of miR-486-5p of CSC Phenotype has Diagnostic and Prognostic Potential in Colorectal Cancer

Andrea Pisano [1,2,3,†], Carmen Griñan-Lison [3,4,5,†], Cristiano Farace [1,2,†], Giovanni Fiorito [1,6], Grazia Fenu [1], Gema Jiménez [3,4,5,7], Fabrizio Scognamillo [8], Jesùs Peña-Martin [3,4,5,7], Alessio Naccarati [9,10], Johannes Pröll [11,12,13], Sabine Atzmüller [12], Barbara Pardini [9,10], Federico Attene [8], Gabriele Ibba [1], Maria Giuliana Solinas [1], David Bernhard [14], Juan Antonio Marchal [3,4,5,7,*] and Roberto Madeddu [1,2,*]

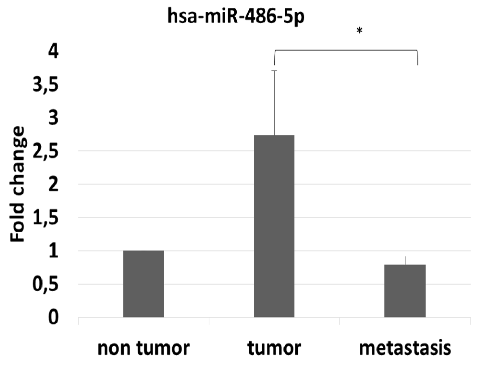

Figure S1A. Comparison between control, tumor CRC and metastatic CRC normalized on hsa-miR-342-3p.

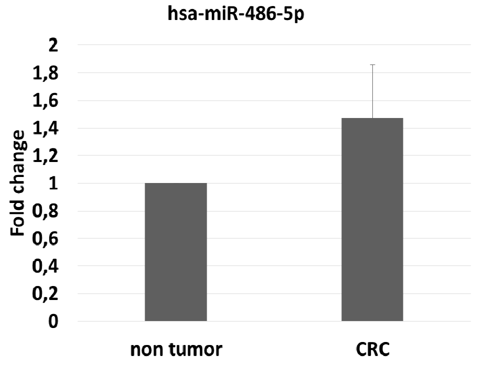

Figure S1B. Comparison between control and CRC normalized on has-miR32-3p.

Table S1. A. p values referred to fig. 5 monolayer.

**Monolayer**			
Stem factor	Control vs mimic	Control vs inhibitor	Mimic vs inhibitor
SOX2	0,18	0,06	0,21
NANOG	0,41	0,07684	0,08
OCT4	<0.01	<0.01	<0.01
KLF4	<0.01	<0.01	<0.01
MYC	<0.01	<0.01	<0.01

(a)

Table S1b. p values referred to fig. 5 CSC.

**CSC**			
Stem factor	Control vs mimic	Control vs inhibitor	Mimic vs inhibitor
SOX2	0,01	<0.01	<0.01
NANOG	0,01	<0.01	<0.01
OCT4	0,81	<0.01	<0.01
KLF4	0,22	<0.01	<0.01
MYC	<0.01	<0.01	<0.01

(b)

Diagnostic and prognostic power of miR-486-5p

We performed a ROC-curve analysis to investigate whether the inclusion of mir486-5p expression values, measured in sera, could increase the diagnostic power of a predictive model. Specifically, we compared the ability to discriminate between CRC patients and healthy controls of two nested logistic regression models: the first (called model 0) includes age, sex, smoking history, levels of carcinoembryonic antigen (CEA), and levels of alkaline phosphatase as predictors; the second (called model 1) including the same predictors as model 0 plus mir486-5p.

The area under the ROC curve (AUC) for model 0 was 0.67 (95% confidence interval 0.43 – 0.91, using DeLong method), whereas the AUC for model 1 was 0.74 (95% confidence interval 0.53 – 0.95, see Figure S2.

Although the difference in the prediction performance of the two models was not statistically significant, we observed a slight increase in the AUC for the model including mir486-5p as an additional predictor, suggesting its diagnostic usefulness in combination with other well-known biomarkers.

Further, we have evaluated the prognostic utility of mir486-5p measured in sera of CRC patients via a Cox regression model to evaluate 5-years survival after surgery. The prediction model includes age, sex, tumour stage and grade, CEA, and alkaline phosphatase as covariates. The hazard ratio (HR) comparing CRC patients within the two extreme quartiles of mir486-5p expression was 0.22(95% confidence interval 0.03 – 2.05), suggesting that, although the difference was not statistically significant, lower levels of mir486-5p cloud be associated with a poor prognosis, even in a model which takes into account other well-known prognostic factors like tumour grade and stage and CEA. The Kaplan Meier curve, according with a stratification of CRC patients in four groups based on mir486-5p expression in sera, is displayed in Figure S3.

Although we are aware of the limitations of these statistical analyses performed on small sample size, our results suggest mir486-5p deserves further investigation to understand whether it can become a useful diagnostic and prognostic biomarker, possibly in combination with those currently used in clinical practice.

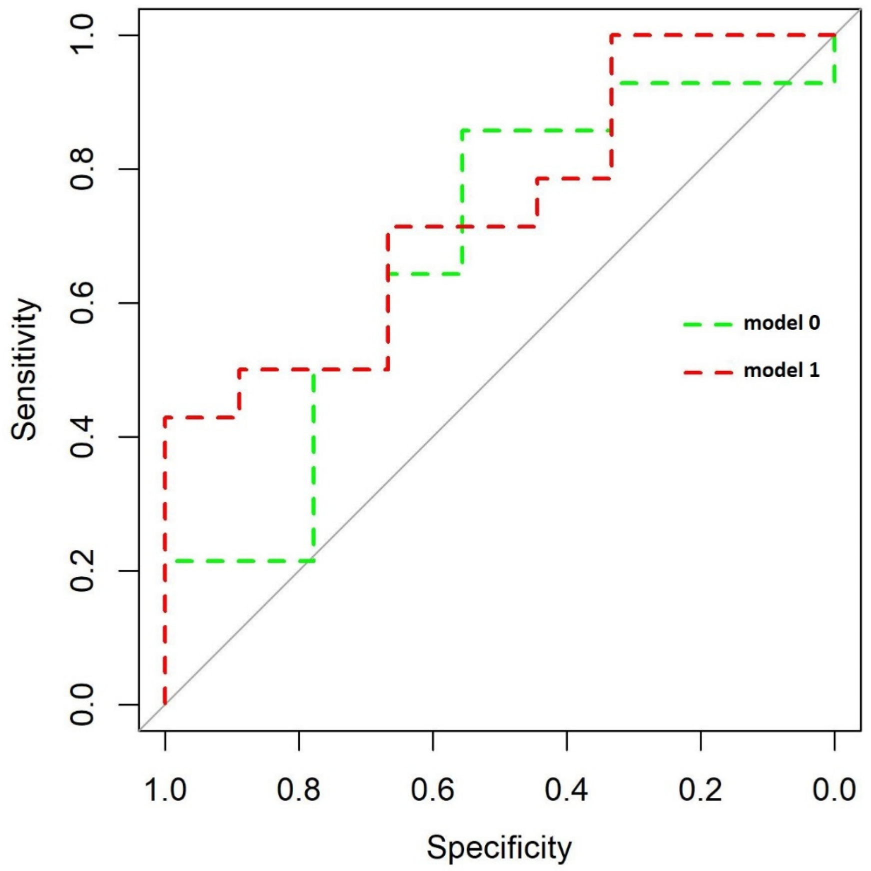

Figure S2. ROC curve of model 0 (not including mir486-5p), in green (AUC = 0.67); and ROC curve of model 1 (including mir486-5p), in red (AUC = 0.74).

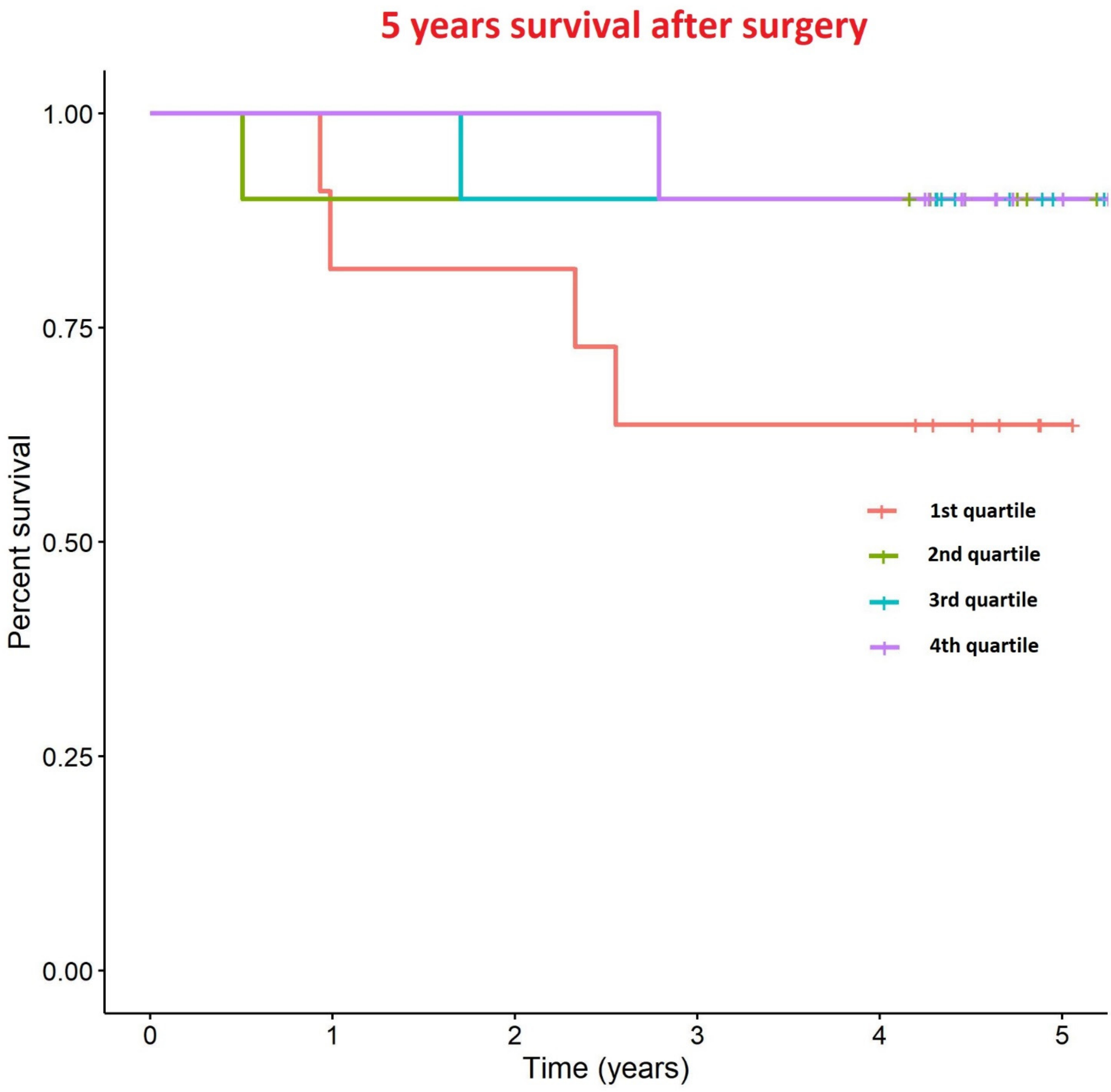

Figure S3. Kaplan Meier plot of CRC patients classified according with mir486-5p expression.

© 2020 by the authors. Submitted for possible open access publication under the terms and conditions of the Creative Commons Attribution (CC BY) license (http://creativecommons.org/licenses/by/4.0/).
